# Suicidality and relation with dissociation and alexithymia in PNES and conversion disorder

**DOI:** 10.1192/j.eurpsy.2021.1576

**Published:** 2021-08-13

**Authors:** L. Longo, T. Jannini, M. Merlo, C. Bombacci, M.R. Biancolino, R. Rossi, E. Di Carlo, C. Niolu, A. Siracusano, G. Di Lorenzo

**Affiliations:** Department Of Systems Medicine, University of Rome Tor Vergata, Rome, Italy

**Keywords:** Psychogenic non-epileptic seizures, Suicidality, PNES, conversion disorder

## Abstract

**Introduction:**

Amongst different subtypes of Conversion Disorder (CD), DSM-V lists the Psychogenic Non-epileptic seizures (PNES). PNES are defined as episodes that visually resemble epileptic seizures but, etiologically, they are not due to electrical discharges in the brain.

**Objectives:**

Our study aims to explore the differences between PNES and other CDs. In particular, we studied the suicidality and its correlations with dissociation and alexithymia.

**Methods:**

Patients, recruited from the Psychiatry and Clinical Psychology Unit of the Fondazione Policlinico Tor Vergata, Rome, Italy, were diagnosed with PNES (n=22) and CD (n=16) using the DSM-5 criteria. Patients underwent the following clinical assessments: HAM-D, BDI, DES, BHS, TAS, CTQ.

**Results:**

PNES showed significantly higher scores than CD in all assessments, except for BDI-somatic (p=0.39), BHS-feeling (p=0.86), and the presence of childhood trauma. PNES also showed significantly higher suicidality (p = 0.003). By controlling for the confounding factor “depression”, in PNES suicidality (and in particular the BHS-loss of motivation) appears to be correlated with DES-total score (p = 0.008), DES-amnesia (p = 0.002) and DES -derealization-depersonalization (p = 0.003). On the other hand, in CDs, the BHS-total score shows a correlation with the TAS-total score (p = 0.03) and BHS-Feelings with TAS-Externally-Oriented Thinking (p = 0.035), while only the BHS-Loss of motivation appears correlated with DES-Absorption (p = 0.011).

**Conclusions:**

Our study shows significant differences between PNES and CD, in several symptomatologic dimensions, including suicidality. Indeed, in PNES suicidality appears to be related to dissociation, while in CDs it appears mainly to be correlated with alexithymia.

